# Evaluation of the Revised Trauma Score, MGAP, and GAP scoring systems in predicting mortality of adult trauma patients in a low-resource setting

**DOI:** 10.1186/s12873-022-00653-1

**Published:** 2022-05-28

**Authors:** Zeinab Mohammed, Yaseen Saleh, Eman Mohammed AbdelSalam, Norhan B. B. Mohammed, Emad El-Bana, Jon Mark Hirshon

**Affiliations:** 1 Department of Public Health and Community Medicine, Faculty of Medicine, Beni-Suef University, Beni-Suef, 62521 Egypt; 2 Department of Emergency Medicine, University of Maryland School of Medicine, Baltimore, MD 21201 USA; 3 Department of Emergency Medicine, University of Miami Health System, Miami, FL 33136 USA; 4 Department of Emergency Medicine, Jackson Memorial Hospital, Miami, FL 33136 USA; 5 Department of General Surgery, Faculty of Medicine, Beni-Suef University, Beni-Suef, 62521 Egypt; 6 Department of Translational Medicine, Translational Glycobiology Institute at FIU, Herbert Wertheim College of Medicine, Florida International University, Miami, FL USA; 7 Department of Medical Biochemistry, Faculty of Medicine, South Valley University, Qena, 83523 Egypt; 8 Department of Orthopedic Surgery, Faculty of Medicine, Beni-Suef University, Beni-Suef, 62521 Egypt; 9 Department of Epidemiology and Public Health, University of Maryland School of Medicine, Baltimore, MD 21201 USA

**Keywords:** Trauma mortality, Trauma scores, Triage, LMICs, Global health

## Abstract

**Background:**

Numerous trauma scoring systems have been developed in an attempt to accurately and efficiently predict the prognosis of emergent trauma cases. However, it has been questioned as to whether the accuracy and pragmatism of such systems still hold in lower-resource settings that exist in many hospitals in lower- and middle-income countries (LMICs). In this study, it was hypothesized that the physiologically-based Revised Trauma Score (RTS), Mechanism/Glasgow Coma Scale/Age/Pressure (MGAP) score, and Glasgow Coma Scale/Age/Pressure (GAP) score would be effective at predicting mortality outcomes using clinical data at presentation in a representative LMIC hospital in Upper Egypt.

**Methods:**

This was a retrospective analysis of the medical records of trauma patients at Beni-Suef University Hospital. Medical records of all trauma patients admitted to the hospital over the 8-month period from January to August 2016 were reviewed. For each case, the RTS, MGAP, and GAP scores were calculated using clinical data at presentation, and mortality prediction was correlated to the actual in-hospital outcome.

**Results:**

The Area Under the Receiver Operating Characteristic (AUROC) was calculated to be 0.879, 0.890, and 0.881 for the MGAP, GAP, and RTS respectively, with all three scores showing good discriminatory ability. With regards to prevalence-dependent statistics, all three scores demonstrated efficacy in ruling out mortality upon presentation with negative predictive values > 95%, while the MGAP score best captured the mortality subgroup with a sensitivity of 94%. Adjustment of cutoff scores showed a steep trade-off between optimizing the positive predictive values versus the sensitivities.

**Conclusion:**

The RTS, MGAP, and GAP all showed good discriminatory capabilities per AUROC. Given the relative simplicity and potentially added clinical benefit in capturing critically ill patients, the MGAP score should be further studied for stratifying risk of incoming trauma patients to the emergency department, allowing for more efficacious triage of patients in lower-resource healthcare settings.

**Supplementary Information:**

The online version contains supplementary material available at 10.1186/s12873-022-00653-1.

## Background

In the management of trauma patients, time is of the essence. Providing appropriate care earlier has been shown to consistently decrease mortality and morbidity [1]. Such prompt care relies on effective and efficient risk stratification in an emergent care setting. Currently, there are numerous trauma scoring systems with varying levels of accuracy and reliability that have been developed for risk classification of morbidity and mortality in incoming trauma patients [2]. These trauma scoring systems have been predominantly used in developed countries for a multitude of uses. Their ability for prognostication has inherent implications for prospective use in triage (though this needs to be coupled with feasibility) as well as retrospective use as measures of injury severity which can be used in quality improvement (QI) projects by comparing actual management and eventual outcomes to expected management and outcomes. In either usage case, the scores need to be validated first as effective discriminators of mortality. Research in both these domains has been particularly deficient in low- and middle-income countries (LMICs) with just five countries (South Africa, Nigeria, India, Iran, and Malaysia) being responsible for the majority of studies [3]. Such studies have generally been done in higher-resourced hospital settings with more robust electronic medical records, equipment, and triage capacities. This is of particular importance as there are wide discrepancies in the conditions seen in the healthcare systems of different LMICs. The present study, though smaller in size, is more representative of conditions seen in lower-resource settings.

The Injury Severity Score (ISS) and Trauma and Injury Severity Score (TRISS) are examples of two well-established scoring systems that have been commonly used in high-income countries to assess the status of trauma patients and to predict the likelihood of survival [4–6]. The ISS depends exclusively on anatomical factors of injuries, while the TRISS synthesizes the mechanism of injury, physiological factors, and anatomical factors. However, despite the efficacy of both these scores, they cannot be feasibly applied to patients upon primary survey at arrival in the emergency department. Calculating these scores requires time and details that are not initially available nor practical for a majority of patients presenting with major trauma [7]. Because of this, it is recommended to calculate the TRISS or ISS within the 24 h *after* trauma admission, limiting their potential for triage utility [8].

Another caveat is that the practice setting can alter which parameters are most suitable for predictive measures. For instance, resource-deficient settings that often hamper hospitals in LMICs frequently lack detailed injury records, protocols, as well as radiographic capabilities. Accordingly, physiologically-based scores like the Revised Trauma Score (RTS) have been discussed as the most pragmatic and effective scoring systems in such settings rather than anatomically-based scores like the ISS or combined scores like the TRISS [3, 9]. In contrast to its anatomically-based counterparts, the RTS can be quickly calculated using a patient’s initial clinical status and vital signs at presentation [10]. Herein lies the importance of validating simplified trauma scores that can predict outcomes in the first “golden hour” upon arrival. Critically, these scores can also be easily applied in low-resource settings that commonly exist in LMICs.

MGAP and GAP are two other predominantly physiologically-based scores that have been validated in research but have yet to be commonly used in low- and middle-income areas despite their promise and feasibility. MGAP is an acronym synthesizing the mechanism of injury (M), the Glasgow Coma Scale [GCS] score (G), the patient’s age (A), and the systolic blood pressure [SBP] (P). It has been validated previously in France to predict 30-day mortality [11]. The GAP score is simplified from the MGAP score by omitting the mechanism of injury and was validated in a sample of trauma patients pulled from Japan’s National Trauma Bank [12]. These two scores differ from the RTS by neglecting the respiratory rate (RR) as well as the score adjustment for head injuries. Notably, calculating MGAP and GAP scores is much easier to do manually than the RTS, which uses a more complex system of coefficients and category codes. The aim of this study was to calculate the RTS, MGAP, and GAP scores for retrospectively pulled adult trauma cases from a representative LMIC hospital in Beni-Suef, Egypt and to compare the correlation between each score’s prediction and the actual in-hospital mortality.

## Methods

### Study design

This study was a retrospective cohort analysis of the medical records of all adult trauma patients admitted to the Beni-Suef University Hospital over the 8-month period from January to August 2016. The results are reported following the STARD (STAndards for the Reporting of Diagnostic accuracy studies) guidelines. The full checklist is available as Supplemental Table 1.

### Setting

Beni-Suef University Hospital is an urban, tertiary-care hospital and the third-largest university hospital in Upper Egypt. It is located at the junction point of Upper and Lower Egypt and serves approximately 3.1 million inhabitants, being the primary care-point of both urban and rural residents in the area. The hospital has 423 beds and 8 intensive care units (ICU). It has an emergency unit that receives all medical and surgical emergencies for both adult and pediatric populations. Furthermore, it is the only hospital in the governorate that is equipped and staffed to provide care for patients with severe trauma. It should be noted that despite the enormous population burden, the Beni-Suef governorate is among the least privileged governorates in Egypt in terms of average income, educational attainment, and funding [13]. The hospital lacks an electronic medical registry and is generally representative of lower-resource settings that are commonly seen in many LMICs.

### Participants and data sources

Trauma was operationally defined using the National Institute of Occupational Safety and Health’s (NIOSH) definition of “an injury or wound to a living body caused by the application of external force or violence” [14]. The decision to admit trauma patients to the Beni-Suef University Hospital is determined collectively by a designated trauma team and on-call physician after assessing the severity of the patient’s physiologic status and the mechanism of injury. For this study, all records of trauma patients that were subsequently admitted were reviewed in order to identify adult patients (> 16 yrs) who met at least one of the following eligibility criteria: (1) inpatient admissions (> 24 h) due to trauma (comprising any mechanism of injury including but not limited to traumatic brain injuries, spinal cord injuries, drownings, and burns patients), (2) transferred trauma patients from other local hospitals, (3) deaths attributable to trauma, or (4) patients requiring trauma team activation/consult. The cutoff for adulthood was set at 16 years of age, as has been previously done (2), as this is a common age in Egypt for increased involvement with motor vehicles (with motor vehicle collisions being a primary cause of trauma in the region), while these patients also physiologically resemble adults, thus preventing a significant impact on subsequent scoring that can occur with younger patients due to differing ranges of normal vital signs and adaptive responses.

Records of patients who were transferred to other hospitals were excluded as well as records lacking the necessary data for the study analyses. Data extraction was done manually from paper medical records as there is no electronic trauma registry available.

### Variables

The data collected consisted of patient demographics, mechanism of injury, SBP, RR, GCS score, in-hospital mortality outcomes, any surgical interventions, and the state of discharge from the hospital. Mechanism of injury was further categorized into blunt trauma (e.g., fall, motor vehicle collision [MVC]) or penetrating trauma (e.g., gunshot, stabbing). The MGAP and GAP scores were then calculated using the standardized scoring system shown in Table 1. Total scores can range from 3 to 29, with a higher score predicting a better prognosis. RTS scores were calculated using an online tool to convert the GCS score, SBP, and RR into the appropriate category code values. RTS scores are also inversely associated with prognosis.

**Table 1:** The GAP and MGAP scoring systems and the corresponding points for each variable (mechanism of trauma is omitted for GAP scores)

Variable	Points Allotted
**Age**	
< 60 years	+ 5
> 60 years	0
**GCS Score**	+ 3–15
**Mechanism of trauma**	
Blunt trauma	+ 4
Penetrating trauma	0
**Systolic blood pressure**	
> 120 mmHg	+ 5
60—120 mmHg	+ 3
< 60 mmHg	0

### Statistical methods

The collected data were tabulated, coded, and analyzed using SPSS for Windows, version 24. Continuous variables were presented as mean values ± standard deviation (SD), and categorical variables were presented as percentages. To test the normality of the calculated MGAP scores, the Kolmogorov–Smirnov test (K-S test) was used. The Mann–Whitney U-test was also used to compare the mean scores in the stratified groups. Finally, the Area Under the Receiver Operating Characteristic curve (AUROC) was used as a measure of predictive performance. DeLong’s test was then used to compare the calculated AUROCs to each other. The level of significance for all the analyses was set at *p* < 0.05.

### Ethics

The study was approved by the Research Ethics Committee at Beni-Suef University and the Institutional Review Board (IRB) at the University of Maryland, USA.

## Results

**Fig. 1:**
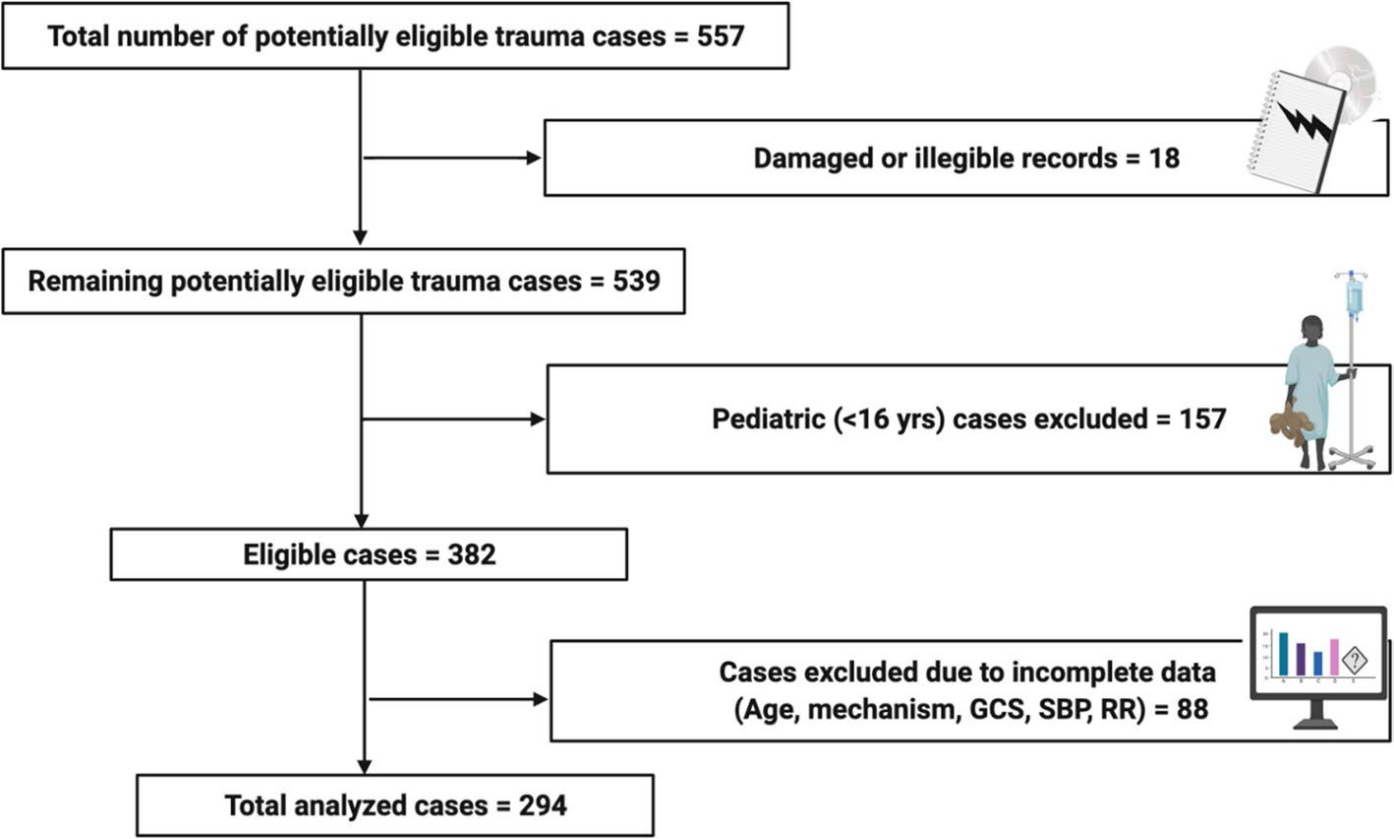
Flow diagram of included and excluded trauma records for analysis

A total of 557 trauma cases were admitted to the hospital during the study period from January 1^st^ to August 31st, 2016. Out of the total records, 18 records were torn or illegible and were accordingly excluded from further analysis. Of the 539 remaining trauma cases, we further excluded 157 cases that were under the age of 16 years. Also excluded were 88 cases that lacked the required data needed to calculate the studied scores such as age, mechanism, GCS, SBP, or RR. The remaining 294 cases (73.5% of the intact, legible cases) matched the criteria and were used for further analysis to compare the three trauma scores (Fig. 1).

### Patients’ demographics

Delving into the patients’ demographics, the mean age was 37.12 ± 16.43 years. 80.3% of the patients were male, while only 19.7% were female. The majority were found to be urban residents (65.3%). Slightly over half of the patients were transported to the hospital by ambulance (53.1%). Finally, only a minority (22.4%) of the patients had established co-morbidities such as diabetes, hypertension, etc. (Table 2).

**Table 2:** Composite demographics and background data of the admitted trauma patients

Demographic variables	Statistical measurement	Number of cases (Total = 294) or Avg. value
**Age (years)**	Mean ± SD	37.14 ± 16.43
**Sex**		
Male	N (%)	236 (80.3)
Female	N (%)	58 (19.7)
**Residence**		
Urban	N (%)	192 (65.3)
Rural	N (%)	102 (34.7)
**Mode of transportation to the hospital**		
Ambulance	N (%)	156 (53.1)
Other	N (%)	138 (46.9)
**Co-morbidities?**		
Yes	N (%)	66 (22.4)
No	N (%)	228 (77.6)

### Clinical data and outcomes

The analyzed records comprised 252 (85.7%) blunt trauma cases and 42 (14.3%) penetrating trauma cases. About half of the cases (50.2%) involved multiple injuries. Vital signs upon initial survey of the patients were also recorded and averaged during analysis (SBP: 112.02 ± 22.5 mmHg, pulse: 112.02 ± 22.5 beats per minute [BPM], and RR: 17.56 ± 5.37 breaths per minute [BrPM]).

Using relevant clinical data from the primary survey, the RTS, GAP, and MGAP scores were calculated for each case. The mean RTS was calculated to be 7.30 ± 1.16, the mean GAP score was 21.10 ± 3.74, and the mean MGAP score was 24.52 ± 4.13. Subsequent clinical care and outcomes were then followed. A total of 60.0% of the cases required activation of the trauma team. The majority (72.1%) of patients then required operative management, while a significant minority (29.3%) required admission to the intensive care unit. Overall, the mortality rate was 18.0% (Table 3). Patients that survived had significantly higher RTS, MGAP, and GAP scores (Table 4). Stratified into subgroups, the mortality rate among patients arriving via ambulance was 25% compared to 10% for patients arriving via other means of transportation (*p* < 0.01). Only the RTS detected a statistically significant difference between the ambulance and civilian transport subgroups; however, the difference was not clinically significant (Supplemental Reviewer Responses).

**Table 3:** Overall clinical data and outcomes of admitted trauma patients

Clinical variables	Statistical measurement	Number of cases (Total = 294) or Avg. value
**Initial vital signs**		
SBP (mmHg)	Mean ± SD	112.02 ± 22.5
Pulse (BPM)	Mean ± SD	80.39 ± 17.45
RR (BrPM)	Mean ± SD	17.56 ± 5.37
**Mechanism of Injury**		
Blunt	N (%)	252 (85.7%)
Penetrating	N (%)	42 (14.3%)
**Polytrauma?**		
Yes	N (%)	152 (51.7)
No	N (%)	142 (48.3)
**Trauma Scores**		
RTS	Mean ± SD	7.30 ± 1.16
GAP	Mean ± SD	21.10 ± 3.74
MGAP	Mean ± SD	24.52 ± 4.13
**Trauma Team Activation**		
Yes	N (%)	194 (66.0)
No	N (%)	100 (34.0)
**Definitive management**		
Operative	N (%)	212 (72.1)
Non-Operative	N (%)	82 (27.9)
**Admission to ICU**		
**Yes**	N (%)	86 (29.3)
**No**	N (%)	208 (70.7)
**Status at discharge**		
Dead	N (%)	53(18.0)
Living	N (%)	241(82.0)

**Table 4:** Clinical data of trauma patients that ultimately survived to discharge compared to non-survivors

Clinical variables	Survivors (*n* = 241)	Non-survivors (*n* = 53)	*P* value
Age (years)	36.05 ± 16.28	42.11 ± 16.33	0.015
SBP (BPM)	116.75 ± 19.59	90.52 ± 22.92	< 0.001
RR (BrPM)	18.08 ± 4.55	15.21 ± 7.73	< 0.001
Pulse	83.73 ± 13.36	66.08 ± 24.50	< 0.001
GCS score	14.06 ± 1.77	8.85 ± 3.84	< 0.001
RTS	7.66 ± 0.47	5.67 ± 1.63	< 0.001
MGAP score	25.72 ± 2.64	19.05 ± 5.17	< 0.001
GAP score	22.22 ± 2.39	16.03 ± 4.52	< 0.001

### AUROC and predictive statistics of the respective scoring systems

**Fig. 2:**
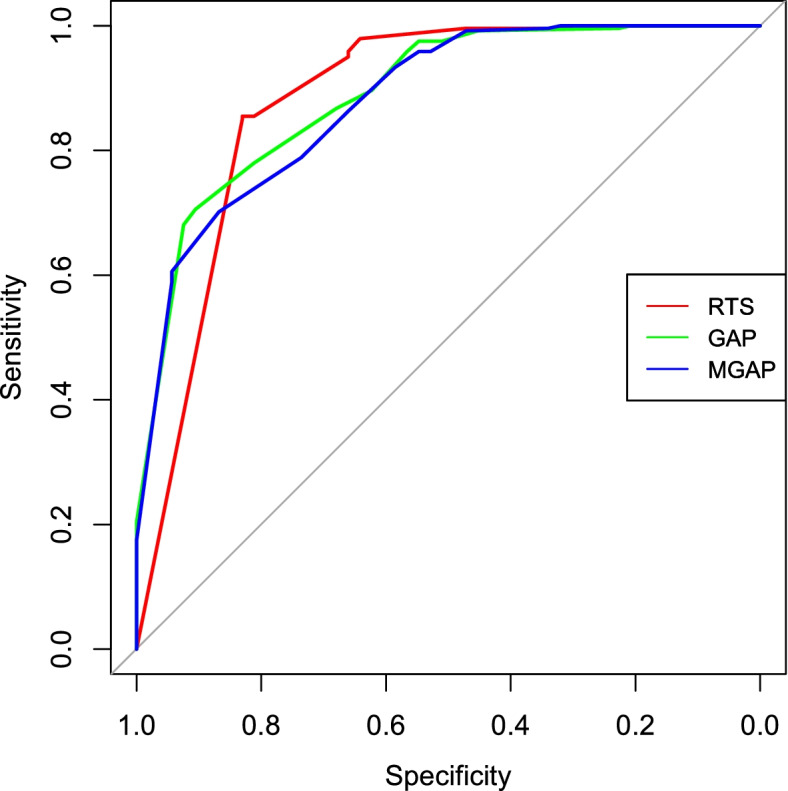
Receiver operating characteristic curve constructed for the respective scores

AUROCs were calculated as a prevalence-independent measure of discrimination to evaluate the RTS, GAP, and MGAP systems in predicting in-hospital mortality as an outcome (Fig. 2). AUROC values for mortality outcomes were comparable at 0.881 (95% Confidence Interval: 0.817–0.945), 0.890 (95% CI: 0.842–0.937), and 0.879 (95% CI: 0.829–0.929) for the RTS, GAP, and MGAP scores respectively with *p*-values unanimously less than 0.001 (Table 5). There were no statistical differences detected between the AUROCs of the three scores using DeLong’s test (Table 6).

**Table 5:** Area under the receiver operating characteristic curve (AUROC) for the three studied scores as well as sensitivities, specificities, and predictive values for sample cutoffs in predicting in-hospital mortality

	**AUROC (95% Confidence Interval)**	***P* Value**	**Cut-off Values for Predicting Mortality**	**Sensitivity (%)**	**Specificity (%)**	**Positive Predictive Value (%)**	**Negative Predictive Value (%)**
**RTS**	0.881 (0.817–0.945)	< 0.001	< 5.7	43	100	96	89
			< 7	83	85	56	96
**GAP**	0.890 (0.842–0.937)	< 0.001	< 15	45	99	92	89
			< 21	81	78	45	95
**MGAP**	0.879 (0.829–0.929)	< 0.001	< 19	47	99	93	90
			< 26	94	61	35	98

**Table 6:** Comparative analysis between the respective AUROCs of the three studied scores using the Delong test

	**RTS vs. GAP**	**RTS vs. MGAP**	**GAP vs. MGAP**
*P* Value	0.70	0.94	0.39

Prevalence-dependent statistics were additionally calculated to enhance the clinical utility of the study (Table 5). The use of more liberal (i.e. higher scores indicative of less physiological derangement) cutoff values such as has often been used in other studies led to the optimization of sensitivities (MGAP > GAP = RTS) with all three scores critically showing excellent efficacy for ruling out trauma mortality (NPVs of 96%, 95%, 98% for the RTS, GAP, and MGAP scores respectively). However, partially due to the lower specificities, the efficacies for positively identifying mortality were roughly 50% (PPVs of 56%, 45%, 35% for the RTS, GAP, and MGAP scores respectively). In contrast, the use of more conservative cutoff values (RTS < 5.7, GAP < 15, MGAP < 19) led to the optimization of both prevalence-dependent predictive statistics with all three scores > 89%. However, here the sensitivities suffered at ~ 50%, meaning a significant portion of the non-survivor subgroup would be falsely negative.

## Discussion

Trauma scoring systems are prominently used to rapidly determine injury severity in order to facilitate triage and predictions of prognosis [12]. The aim of this study was to evaluate the prognostic abilities of the simpler GAP and MGAP scores as compared to the more complex RTS in predicting trauma mortality in a resource-deficient emergency department in a LMIC hospital. Validating the discriminatory ability of these scores will enable future studies on their utility for both prospective triage as well as retrospective QI projects.

Thus far, the primary discussion has focused on the strengths of the RTS, including its potential in maximizing time efficiency. However, the RTS has potential weaknesses that have hindered its implementation. Firstly, its use can result in false negatives in cases of severe injury in a single body area [15]. It additionally neglects the impaired bodily resilience associated with aging. For these reasons, the other studied scores (MGAP and GAP) have been advocated by researchers to meet these flaws. As previously noted, the MGAP scoring system has been validated by a study in France [11] due to the critical importance of validation of these scores prior to application in clinical practice to prevent adverse outcomes [16]. Sartorius et al. expanded on this characterization by demonstrating the MGAP system can clearly outline the differences in mortality outcomes between low-, intermediate-, and high-risk groups, even more specifically than the Triage-Revised Trauma Score (T-RTS), RTS, and TRISS [11]. Like the TRISS, the MGAP score incorporates two anatomic components that distinguishes it from the RTS. The first is the mechanism of trauma, which helps to cover the largest subset of false negatives produced by the RTS [17, 18]. The second component is age. Age is considered an important factor in predicting mortality, which is significantly higher among the elderly, who often have weakened adaptive responses [19, 20].

Another area of the trauma score discussion that requires further exploration (especially in low-resource settings) is the concept of resource allocation, as this can be a major hinderance to feasibility. An example of this is the New Trauma Score (NTS) introduced by Jeong et al. in 2017, which improved on the RTS by using peripheral oxygen saturations (SpO2) instead of the RR as well as modified point values for the GCS and SBP aspects [21]. Despite its predictive success, however, the MGAP and GAP scores were found to be superior to the NTS in more than one aspect with respect to application in low resource settings. For instance, the NTS depends on measuring the patient’s SpO2; however, pulse oximeters are often not available upon initial presentation to the emergency department in such settings, being reserved for the ICU (if available). Likewise, the RTS similarly suffers from a reliance on accurate measurement of a patient’s RR, which at the time of a trauma code may require similar equipment. Accordingly, the MGAP and GAP scores can be more feasibly and accurately calculated for trauma patients in modest-resource trauma centers and critically for patients *at the time of presentation* rather than at a delayed timepoint [21]. Jeong et al. subsequently concluded that the NTS is better than the RTS but fails to overtake the efficacy and efficiency of the MGAP and GAP scores.

Our findings were consistent with the literature. The patient population was found to be representative of the international trauma epidemic, with younger males known to be disproportionately affected at 2–3 times the rate of females (Table 2). Patients that ultimately survived showed higher average RTS, MGAP, and GAP scores, accurately representing stabler vital signs and likely better overall condition (Table 4). Most importantly, the data revealed all three scores to have good AUROC values (0.881 for the RTS, 0.890 for the GAP score, and 0.879 for the MGAP score), demonstrating efficacy as a predictive measure. There were no statistical differences detected between the scores using DeLong’s test. These results were consistent with those reported previously by Ahun et al. and Jeong et al. [7, 21]. Importantly, these results were also consistent with a similar study in another LMIC setting (Mumbai, India), where the authors calculated AUROC values of 0.85, 0.85, and 0.84 for the RTS, GAP, and MGAP scores respectively, which may suggest broader applicability [22].

Evaluating the prevalence-dependent statistics in particular additionally generated several points of discussion regarding clinical utility. The use of more liberal score cutoff values resulted in excellent negative predictive values above 95%, demonstrating efficacy in the ability to rule out mortality in a low-resource setting. In particular, the MGAP score was especially adept at capturing almost the entirety of the mortality subgroup with a sensitivity of 94%. The more liberal cutoff values, however, presented a secondary issue in that with PPVs at around 50%, a large quantity of resources may potentially be diverted to a significant volume of patients mischaracterized from the survivor subgroup. A confounding factor here is that many of these “false positives” showed lower scores due to significant physiological derangement and morbidity that required ultimately successful intervention in the ICU. Accordingly, this probably represents a desirable manner of both ruling out severe outcomes *and* accurately identifying critically ill patients. In contrast, usage of more conservative cutoff scores optimized the predictive values of the scores (perhaps improving resource efficiency); however, this resulted in close to 50% of the mortality subgroup being missed as false negatives, which is especially problematic given the high mortality rate (18%) of the studied trauma population. Overall, considering the advantage that it can be reasonably and accurately applied to the evaluation of patients *at presentation* while also maintaining the best sensitivity with comparable predictive values, use of the MGAP score could robustly improve the ability to triage in LMICs with a look to reducing morbidity and mortality in a cost-effective manner. This aspect is especially important in high volume, low-resource environments, where many critically ill patients may be missed by physicians due to time and attention constraints.

Despite the implicated potential for prognostic-based triage, the implementation of these scoring systems in LMIC trauma care should be conceived cautiously with frequent quality assessment. Many studies have highlighted the importance of the motor component of the GCS score for prognosis. However, the absence of specific GCS details in a large percentage of trauma records prevents the evaluation of this hypothesis [23]. Ultimately, the efficacy of such scoring systems for prospective use in triage would depend on the reliability of these specific components as well as the time at which these scores are determined. Ideally, such scores could be calculated in the pre-hospital stage; however, in locations similar to Egypt, this may not be feasible due to the lack of trained ambulatory staff and electronic communication with the hospital. Accordingly, these scores would likely need to be determined after the application of initial Advanced Trauma Life Support (ATLS) guidelines, which may affect their utility in affecting disposition, though they can still serve as an evidence-based measure to buttress care plans.

Additionally, some limitations of the study should be mentioned. Firstly, this study was a retrospective study, which is inherently subject to more confounding variables than a prospective study which can be more standardized. Specifically, the data analyzed are limited to that which is already recorded in the existing registry. Here, a considerable number of records (23% of the eligible cases) required exclusion as they simply lacked the required data (GCS scores, vitals, etc.) needed to calculate the scores in the study. Of note, this figure was substantially lower than that of similar studies in other LMICs where the RTS could not be calculated in 65–98% of cases retrospectively, likely reflecting local differences in documentation [3]. Even so, this exposes the study to a sampling bias since, for instance, it may be that the assessing physicians only felt the need to record certain data points if they were critical to the patient’s management. The lack of uniform guidelines for triaging patients in the hospital could also affect the quality of medical care delivered to the patient, potentially skewing mortality statistics.

Retrospective studies additionally preclude certain analyses. For example, the Kampala Trauma Score (KTS) has been proposed as a potential triage tool, showing efficacy as a discriminator of trauma mortality in other resource-limited settings [3]. However, the score relies on an assessment of neurological status that could not be determined retrospectively in this study, as only total GCS scores are recorded in the existing records. In a similar vein, the lack of temperature data (found in only 22.1% of the records) precluded the study of the Worthing Physiological Scoring system, which was found to be to be superior to the RTS in predicting both mortality and morbidity in another study in Iran, though both scores still showed good discriminatory capacity [24]. Furthermore, the records used do not contain documentation of the ISS nor all the required information to calculate it. Thus, we were unable to use the ISS as an intermediate point, which has been used in many similar studies. Due to the established robustness of the ISS, other studies have often tested new scores’ mortality prediction abilities against the ISS. The lack of ISS data accordingly prevented further validation here. Finally, the pediatric population was excluded, so the findings reported are only applicable to adults.

Another limitation of trauma assessment in general lies in the variability in measuring vital signs. In the hospital setting, there is an intra-observer variability of measuring the cardiac pulse of up to 10–15%, SBP of up to 20–25%, and RR by more than 30% where these are not measured electronically. It is accordingly expected that the reproducibility of trauma scores that incorporate vital signs strongly depends on the reliability of their measurement [25]. This reliability can falter in low-resource settings due to reasons such as low staff-to-patient ratios, dysfunctional equipment, and disorganized emergency rooms. Similarly, the data collected in this study comes from one study center, making the generalizability of the findings limited to settings with similar conditions as described above. Specifically, predictive value statistics depend on the prevalence of the tested condition. Accordingly, variances in this can affect the applicability of these results.

Variances in the study cohort compared to other regions can also affect general applicability. Given the relative youth of the cohort, it was not surprising that the rate of co-morbidities was low in this study (22.4%), though in a low-resource setting this can reflect a lack of adequate primary care. It should be noted, however, that in populations with a higher prevalence of co-morbidities, predictive statistics established here may be less applicable given the increased propensity in these patients for rapid deterioration. Additionally, 85.7% of the cases in this study were blunt trauma cases, which tends to be more prevalent in the majority of areas internationally. However, in areas with higher incidents of penetrating trauma (such as areas marred by gun violence), the discriminative ability of the scores used here may also be less applicable.

Moving forward, future studies are required to further enhance the discussion. The implementation of prospective studies to validate these scores can help to reduce the confounding variables of a retrospective study, as the data collected can be standardized. Such studies would additionally allow for assessment of the practicality of using scores for triaging decisions in real-time, which is possibly the most important characteristic required of a trauma score in a high-volume, low-resource setting. For instance, one study validated the KTS as a retrospective classifier of injury but found the predictive value may not be strong enough to merit use as a triage tool [26]. From the retrospective angle, an important area of research that requires further investigation is the implementation of these trauma scores as part of a QI process, as there is a dearth of literature available from LMICs [3]. Finally, it would be beneficial to assess these scores at multiple study centers across the region to enhance the generalizability of the results.

## Conclusion

Trauma scores are valuable tools in predicting patient prognosis, thus facilitating initial triage. The MGAP score is one such score that effectively combines anatomical and physiological data, while maintaining time- and cost-efficient feasibility in low-resource settings, which may not be as possible with the RTS. Here, the MGAP, GAP, and RTS scores were all demonstrated to have an excellent ability in ruling out in-hospital mortality. The MGAP score in particular captured patients with subsequent mortality the best while still maintaining comparable clinical predictive values (and thus resource efficiency). In light of this, we recommend maximizing its study for pre-hospital and ED triage, care evaluation, and injury research due to its simplicity and likely feasibility. Doing so has the potential to vastly improve trauma care in regions with known limited resources or triage systems that are not evidence-based, as is common in many areas in low- and middle-income countries like Egypt.

## Supplementary Information

**Additional file 1:Supplemental Table 1.** STARD Guidelines Checklist (obtained from the EQUATOR Centre).

## Data Availability

Local governmental and institutional guidelines prevent the public storage of raw patient health data. However, the datasets generated during and/or analyzed during the current study are available from the corresponding author on reasonable request.
